# Interaction of γ-Polyglutamic Acid/Polyethyleneimine/Plasmid DNA Ternary Complexes with Serum Components Plays a Crucial Role in Transfection in Mice

**DOI:** 10.3390/pharmaceutics16040522

**Published:** 2024-04-09

**Authors:** Tomotaka Ko, Shintaro Fumoto, Tomoaki Kurosaki, Moe Nakashima, Hirotaka Miyamoto, Hitoshi Sasaki, Koyo Nishida

**Affiliations:** 1 Graduate School of Biomedical Sciences, Nagasaki University, 1-7-1 Sakamoto, Nagasaki 852-8501, Japan; 2 Institute of Tropical Medicine, Nagasaki University, 1-12-4 Sakamoto, Nagasaki 852-8523, Japan

**Keywords:** in vivo gene delivery, γ-polyglutamic acid, plasmid DNA, anionic polyplexes, serum components

## Abstract

Typical examples of non-viral vectors are binary complexes of plasmid DNA with cationic polymers such as polyethyleneimine (PEI). However, problems such as cytotoxicity and hemagglutination, owing to their positively charged surfaces, hinder their in vivo use. Coating binary complexes with anionic polymers, such as γ-polyglutamic acid (γ-PGA), can prevent cytotoxicity and hemagglutination. However, the role of interactions between these complexes and serum components in in vivo gene transfer remains unclear. In this study, we analyzed the contribution of serum components to in vivo gene transfer using PEI/plasmid DNA binary complexes and γ-PGA/PEI/plasmid DNA ternary complexes. In binary complexes, heat-labile components in the serum greatly contribute to the hepatic and splenic gene expression of the luciferase gene. In contrast, serum albumin and salts affected the hepatic and splenic gene expression in the ternary complexes. Changes in physicochemical characteristics, such as increased particle size and decreased absolute values of ζ-potential, might be involved in the enhanced gene expression. These findings would contribute to a better understanding of in vivo non-viral gene transfer using polymers, such as PEI and γ-PGA.

## 1. Introduction

Gene therapy has the potential for causal treatments of intractable diseases, such as cancer and congenital genetic defects [1,2,3]. Advances in gene transfer technologies are essential for the development of novel therapies. Gene transfer methods can be broadly classified as ex vivo and in vivo methods. Although ex vivo methods have fewer ethical issues than in vivo methods, the latter are simple because they require neither specific equipment for cell culture nor selection of desirable cells. As gene transfer methods, viral vectors have high gene transfer efficiency and have been subjected to numerous clinical trials [3]; however, there are several concerns, such as immunogenicity [4,5]. Therefore, expectations from non-viral vectors with low toxicity and easy modification are high [6]. Typical examples of non-viral vectors include complexes of plasmid DNA with cationic polymers or liposomes. In particular, various types of polymers are used for gene delivery, including polyethyleneimine [7,8,9,10,11,12,13,14], poly-β-amino esters [15,16,17,18], chitosan [19,20,21,22], and dendrimers [23,24]. These complexes have been reported to exhibit high gene expression in vitro and in vivo. These complexes are prepared with excess cations over the anionic plasmid DNA. Their positively charged surfaces allow them to easily adhere to negatively charged cell surfaces and efficiently introduce genes into the cells. However, aggregation with erythrocytes is problematic when these complexes are systemically administered [25,26].

Polyethyleneimine (PEI) is a cationic polymer that is used to form complexes with plasmid DNA. Plasmid DNA/PEI binary complexes exhibit high gene expression both in vitro and in vivo [7,10,12,27,28]. The binary complexes bind non-specifically to negatively charged cell surfaces and aggregate with blood components, such as erythrocytes [26]. Aggregation can promote their elimination from the body and cause toxicity, including vascular embolization and inflammatory reactions [29,30].

Several methods have been investigated to reduce cytotoxicity and hemagglutination by coating cationic complexes with anionic substances [31,32,33]. Moreover, we reported the usefulness of an anionic polymer γ-polyglutamic acid (γ-PGA) [34,35]. The ternary complexes of γ-PGA/cationic polymer/plasmid DNA did not induce hemagglutination and resulted in high gene expression in B16-F10 melanoma cells at a level comparable to that of the cationic polymer/plasmid DNA binary complexes. The ternary complexes are effective in mice, particularly in the spleen [35].

It is necessary to understand the in vivo fate of these complexes before their future clinical use. For systemic administration, interactions between blood components and the complexes should be considered. As cationic liposome/plasmid DNA complexes have a positively charged surface, they electrostatically interact with negatively charged biological components such as serum proteins and blood cells. The interaction of these complexes with erythrocyte suspensions causes aggregation [25]. Premixing the complexes with erythrocyte suspensions decreased gene expression in the lungs after intravenous administration in mice, whereas pre-mixing with serum did not decrease gene expression [36]. Based on studies using cultured cells, the interaction between complexes and serum has been considered as an inhibitory factor for transfection using cationic liposome/plasmid DNA complexes [37]. As serum does not inhibit lipofection in vivo [36], there are discrepancies between in vitro and in vivo studies on the role of serum. Therefore, it is important to analyze the role of serum components in gene transfer in vivo. Regarding the effect of serum on PEI-based transfection, similar to liposome-based transfection, serum is considered an inhibitory factor in cell culture conditions [38]. In this study, we analyzed the role of serum components in gene transfer using PEI/plasmid DNA binary complexes and γ-PGA/PEI/plasmid DNA ternary complexes in mice.

## 2. Materials and Methods

### 2.1. Materials

Bovine serum albumin (BSA; fraction V) and PEI (branched; average molecular weight, 25,000) were purchased from Sigma-Aldrich (St. Louis, MO, USA). γ-PGA (linear; average molecular weight, 10,000–100,000) was obtained from Yakult Pharmaceutical Industry Co., Ltd. (Tokyo, Japan).

### 2.2. Animals

Five-week-old male ddY mice were purchased from Kyudo Co., Ltd. (Kumamoto, Japan). They were housed in an air-conditioned room and allowed access to water and a standard laboratory diet (MF, Oriental Yeast, Co., Ltd., Tokyo, Japan) ad libitum. All animal experiments were performed in accordance with the Guidelines for Animal Experimentation of Nagasaki University (approval number: 1304171055). 

### 2.3. Plasmid DNA

Plasmid DNA encoding firefly luciferase was constructed in a previous report [39]. The plasmid DNA was amplified in *Escherichia coli* (DH5α) and purified using an EndoFree Plasmid Giga Kit (QIAGEN GmbH, Hilden, Germany).

### 2.4. Preparation of Binary Complexes

PEI/plasmid DNA binary complexes were prepared according to a previous report [34]. Briefly, plasmid DNA in a sterile 5% glucose solution (30 µg/100 µL) was added to PEI in a sterile 5% glucose solution (31.35 µg/100 µL, pH 7.4), and the mixture was incubated for 15 min at room temperature (25 °C). The molar ratio of PEI to plasmid DNA in the mixture was 8.0 (nitrogen of PEI to phosphate of the plasmid DNA).

### 2.5. Preparation of Ternary Complexes

γ-PGA/PEI/plasmid DNA ternary complexes were prepared according to a previous report [34]. Briefly, plasmid DNA in sterile 5% glucose solution (30 µg/66.67 µL) was added to PEI in sterile 5% glucose solution (31.35 µg/66.67 µL, pH 7.4), and the mixture was incubated for 15 min at room temperature (25 °C). Then, γ-PGA in a sterile 5% glucose solution (70.38 µg/66.67 µL) was mixed with the PEI/plasmid DNA binary complexes, and the mixture was incubated for 15 min at room temperature (25 °C) to produce γ-PGA/PEI/plasmid DNA ternary complexes. The molar ratio of γ-PGA/PEI/plasmid DNA in the mixture was 6.0/8.0/1.0 (carboxylate of γ-PGA/nitrogen of PEI/phosphate of plasmid DNA).

### 2.6. Preparation of Serum Components

Blood was collected using a syringe with a needle from the vena cava of anesthetized mice and allowed to clot by incubation at room temperature (25 °C) for 3 h. The clotted blood was centrifuged (15,000× *g*) for 5 min to obtain serum. The serum was then incubated for 30 min in a water bath (56 °C) to produce heat-inactivated serum. The concentration of BSA in phosphate-buffered saline (PBS) was set to 30 mg/mL as a physiological concentration [40].

To investigate the effects of each component, we removed certain components from serum using ion-exchange chromatography columns. To remove anionic components, equal volumes of serum and starting buffer (20 mM Tris/HCl buffer, 110 mM NaCl, and 2 mM EDTA; pH 7.0) were mixed, and the mixture was applied to an anion-exchange column (Vivapure Q, Sartorius Stedim Biotech, Aubagne, France). The column was centrifuged (2000× *g*, 4 °C, 5 min), and the flow-through fraction was recovered. The buffer of the fraction was replaced with PBS using a desalting column (PD-10; GE Healthcare Life Sciences (currently Cytiva), Marlborough, MA, USA). The sample was then concentrated using a spin column (Vivaspin 500, MWCO 10,000, Sartorius Stedim Biotech), and the volume of the sample was adjusted to the initial volume of serum to produce QF. To remove cationic components, equal volumes of serum and starting buffer (20 mM Tris/HCl buffer, 110 mM NaCl, 2 mM EDTA, pH 7.0) were mixed, and the mixture was applied to a cation-exchange column (Vivapure S, Sartorius Stedim Biotech, Aubagne, France). The column was centrifuged (2000× *g*, 4 °C, 5 min), and the flow-through fraction was recovered. The buffer of the fraction was replaced with PBS using a desalting column (PD-10; GE Healthcare Life Sciences (currently Cytiva), Marlborough, MA, USA). The sample was then concentrated using a spin column (Vivaspin 500, MWCO 10,000, Sartorius Stedim Biotech), and the volume of the sample was adjusted to the initial volume of serum to produce SF.

To investigate the effect of each component, the complexes (200 µL) were incubated with each component (100 µL) for 5 min at 37 °C before administration. For control experiments, the complexes were incubated in a 5% glucose solution.

### 2.7. In Vivo Gene Transfer

The complexes (30 μg of plasmid DNA) with serum components were injected intravenously into mice. Six hours after injection, the mice were sacrificed under anesthesia by cervical dislocation. Each tissue sample was dissected, washed with saline, and cooled on ice. An ice-cooled lysis buffer (0.1 M Tris/HCl buffer, 0.05% Triton X-100, 2 mM EDTA, pH 7.8) was added to each tissue at a ratio of 4 µL/mg tissue. The tissues were homogenized and centrifuged (15,000× *g*, 4 °C, 5 min). The resultant supernatant (20 µL) was mixed with luciferase substrate solution (100 µL), and the produced light was measured using a luminometer (Lumat LB9507, Berthold Technologies, Bad Wildbad, Germany). Luciferase activity was expressed as relative light units (RLU) per gram of tissue.

### 2.8. Physicochemical Characteristics Measurement

The particle size (Z-average), polydispersity index (PDI), and ζ-potential of the complexes with serum components were measured using Zetasizer Pro (Malvern Panalytical Ltd., Worcestershire, UK).

### 2.9. Microscopic Image Analysis

The complexes with serum components (5% glucose (control), serum, heat-inactivated serum, BSA in PBS, and PBS) were observed using an inverted microscope (AxioVert A1, Carl Zeiss Microscopy GmbH, Jena, Germany) with a 20× objective lens (Plan-Apochromat, numerical aperture 0.8, differential interference contrast mode). 

### 2.10. Plasmid DNA Accessibility

To assess the accessibility of plasmid DNA, we performed an intercalation assay using a dsDNA-specific dye. The ternary complexes were prepared as described above. The ternary complexes (20 μL) were incubated with 10 μL of serum components (5% glucose (control), serum, heat-inactivated serum, BSA in PBS, and PBS) for 5 min at 37 °C. Then, 2 μL of the ternary complexes with serum components were mixed with 200 μL of the dsDNA dye (QuantiFluor ONE dsDNA System, Promega Corporation, Madison, WI, USA). Five minutes after incubation with dsDNA dye, accessible plasmid DNA was quantified using a Quantus Fluorometer (Promega Corporation).

### 2.11. Statistical Analysis

Statistical analysis was performed using one-way analysis of variance with Tukey’s post-hoc test.

## 3. Results

### 3.1. Effect of Interaction with Serum on In Vivo Transfection Using Binary Complexes

**Figure 1:**
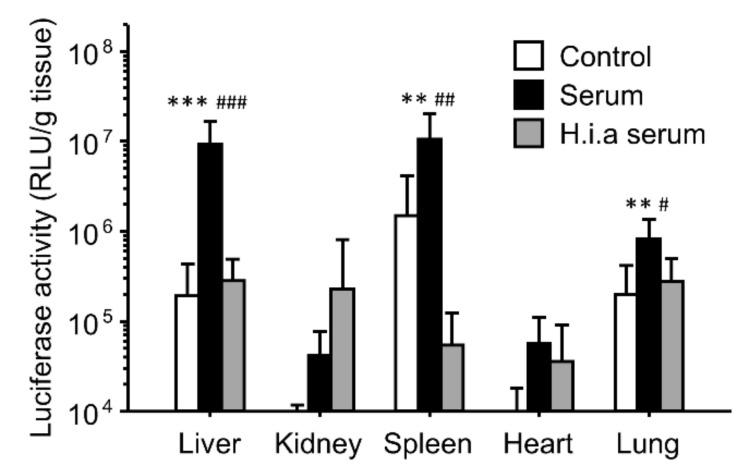
Effect of the interaction with serum on in vivo transfection using PEI/plasmid DNA binary complexes. The binary complexes were incubated with 5% glucose solution (control), serum, or heat-inactivated serum (h.i.a serum) before intravenous injection into mice. Transfection efficiencies 6 h after injection are shown. Each bar represents the mean + S.D. of at least 4 replicates. **, *** indicate statistical differences vs. control (*p* < 0.01, 0.001, respectively). #, ##, ### indicate statistical differences vs. h.i.a. serum (*p* < 0.05, 0.01, and 0.001, respectively).

To investigate the effect of the interaction with serum on in vivo transfection using PEI/plasmid DNA binary complexes, we incubated the complexes with serum or heat-inactivated serum before intravenous injection into mice (Figure 1). Incubation of the complexes with serum before administration greatly enhanced gene expression of the luciferase gene in the liver, spleen, and lung. In contrast, heat inactivation of the serum diminished the enhancing effects of the gene expression.

### 3.2. Effect of Interaction with Serum on In Vivo Transfection Using Ternary Complexes

**Figure 2:**
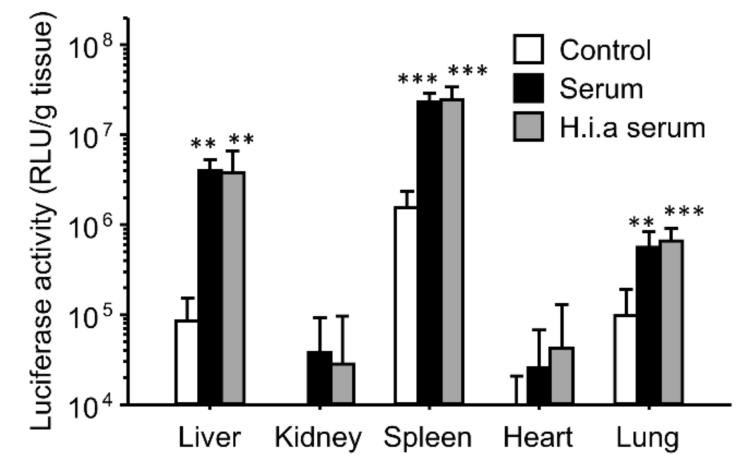
Effect of interactions with serum on in vivo transfection using γ-PGA/PEI/plasmid DNA ternary complexes. The ternary complexes were incubated with a 5% glucose solution (control), serum, or heat-inactivated serum (h.i.a serum) before intravenous injection in mice. Transfection efficiencies 6 h after injection are shown. Each bar represents the mean of +S.D. at least 4 replicates. **, *** indicate statistical differences vs. control (*p* < 0.01, 0.001, respectively).

Furthermore, we investigated the effect of the interaction with serum on in vivo transfection using γ-PGA/PEI/plasmid DNA ternary complexes (Figure 2). Incubation of the complexes with serum before administration greatly enhanced the gene expression in the liver, spleen, and lungs. Although heat inactivation of serum completely diminished the enhancing effects of the binary complexes on the gene expression, it retained the enhancing effects in the ternary complexes.

### 3.3. Effect of Separation of Serum Components on the Enhancing Effects of the Gene Expression for Ternary Complexes

**Figure 3:**
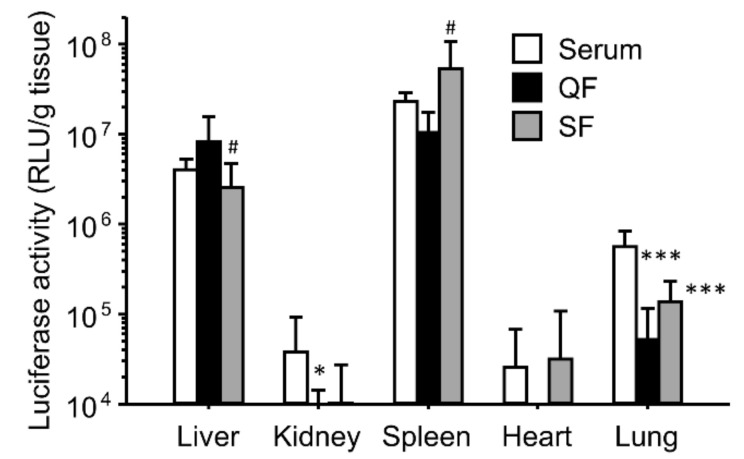
Effect of interactions with separated serum components on in vivo transfection using γ-PGA/PEI/plasmid DNA ternary complexes. The ternary complexes were incubated with serum, anion-exchange column flow through the QF, or cation-exchange column flow through the SF before intravenous injection in mice. Transfection efficiencies 6 h after injection are shown. Each bar represents the mean + S.D. of at least 4 replicates. *, *** indicate statistical differences vs. serum (*p* < 0.05, 0.001, respectively). # indicates statistical differences vs. QF (*p* < 0.05).

Next, we analyzed the serum components that affected the gene expression. Anion- and cation-exchange columns were used to remove the cationic and anionic components from the serum, respectively (Figure 3). Even after removing anionic and cationic components, the gene expression in the liver and spleen did not decrease, whereas the gene expression in the lungs decreased, compared with that in the serum group. Thus, common components of QF and SF may contribute to the enhancement of gene expression.

### 3.4. Effect of BSA and PBS on the Enhancing Effects of the Gene Expression for Ternary Complexes

**Figure 4:**
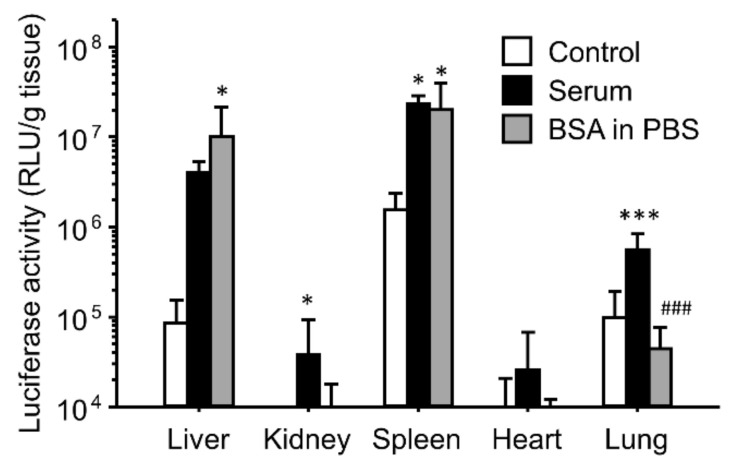
Effect of interactions with BSA on in vivo transfection of γ-PGA/PEI/plasmid DNA ternary complexes. The ternary complexes were incubated with a 5% glucose solution (control), serum, or BSA in PBS before intravenous injection into mice. Transfection efficiencies 6 h after injection are shown. Each bar represents the mean + S.D. of at least 4 replicates. *, *** indicate statistical differences vs. control (*p* < 0.05, 0.001, respectively). ### indicates statistical differences vs. serum (*p* < 0.001).

As serum albumin may be a major component of both QF and SF, we tested the effect of BSA on the enhancing effects of the gene expression (Figure 4). BSA was dissolved in PBS. The enhanced effects of the gene expression in the liver and spleen were reproduced by incubation of the ternary complexes with BSA in PBS, whereas the gene expression in the lungs did not change after incubation with BSA.

**Figure 5:**
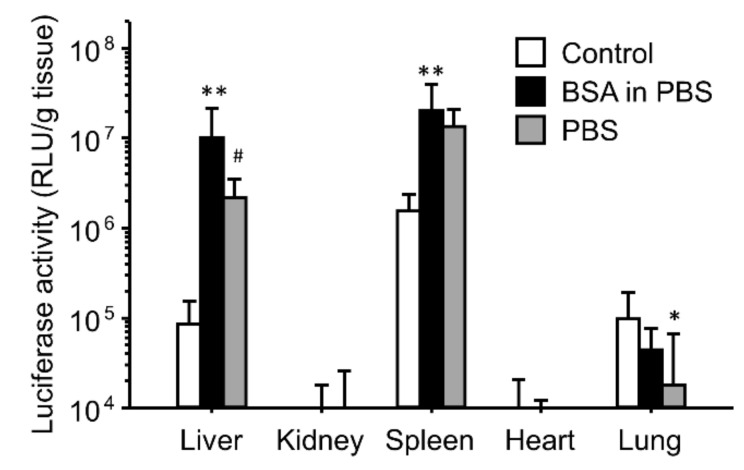
Effect of incubation with PBS on the in vivo transfection of γ-PGA/PEI/plasmid DNA ternary complexes. The ternary complexes were incubated with a 5% glucose solution (control), BSA in PBS, or PBS before intravenous injection into mice. Transfection efficiencies 6 h after injection are shown. Each bar represents the mean + S.D. of at least 4 replicates. *, ** indicate statistical differences vs. control (*p* < 0.05, 0.01, respectively). # indicates statistical differences vs. BSA in PBS (*p* < 0.001).

Subsequently, we focused on the effects of the ionic solutions. The ternary complexes were incubated with phosphate-buffered saline (PBS) as an ionic solution before administration (Figure 5). The enhancing effects of PBS on the gene expression in the liver and spleen were slightly lower than those of BSA in PBS and serum (Figure 4 and Figure 5); however, the gene expression levels in the liver and spleen of the PBS group were higher than those in the control group. Inversely, incubation of the ternary complexes with PBS decreased the gene expression in the lungs.

### 3.5. Effect of Incubation with Serum Components on the Physicochemical Characteristics of the Ternary Complexes

**Figure 6:**
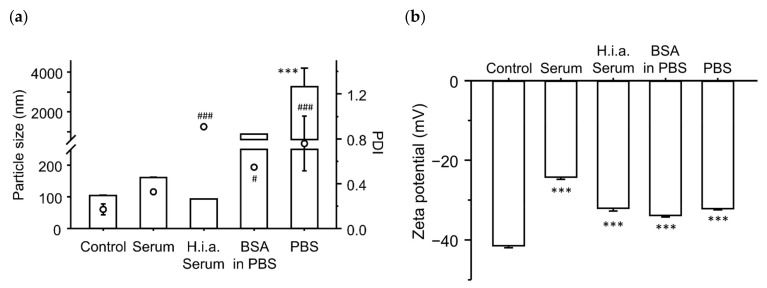
Effect of incubation with serum components on the physicochemical characteristics of the γ-PGA/PEI/plasmid DNA ternary complexes. (**a**) Particle size (bars) and PDI (circle dots). (**b**) ζ-potential. Each bar and dot represent the mean + S.D. of 3 replicates. *** indicates statistical differences vs. control for size and ζ-potential (*p* < 0.001). #, ### indicate statistical differences vs. control for PDI (*p* < 0.05, 0.001, respectively).

Finally, we investigated the effects of incubation with serum components on the physicochemical characteristics of the ternary complexes (Figure 6). Incubation of the ternary complexes increased their particle size and PDI. In the case of heat-inactivated serum, the particle size remained unchanged, whereas PDI increased significantly. In both the BSA in PBS and PBS groups, particle size and PDI increased significantly. Regarding the ζ-potential, incubation of the ternary complexes decreased the absolute values of all groups compared to the control group.

**Figure 7:**
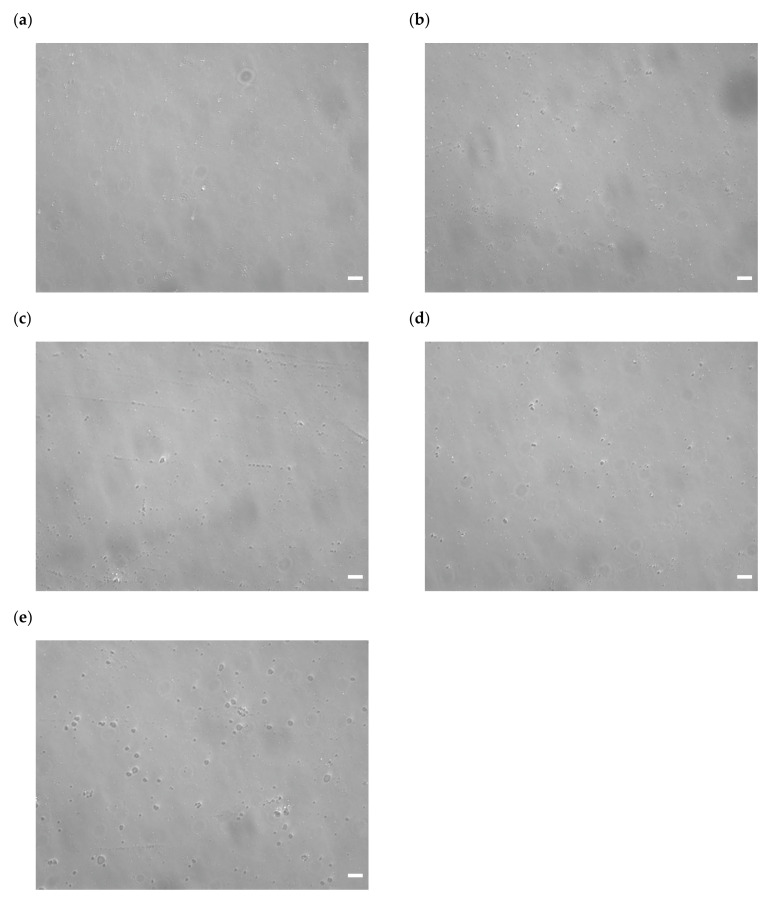
Microscopic images of the γ-PGA/PEI/plasmid DNA ternary complexes after incubation with serum components. The ternary complexes were incubated with (**a**) 5% glucose (control), (**b**) serum, (**c**) heat-inactivated serum, (**d**) BSA in PBS, and (**e**) PBS. Scale bar: 20 μm.

An increase in particle size after the interaction of the ternary complexes with the serum components was also observed by microscopy (Figure 7). In the control group (incubation with 5% glucose), some visible particles were observed, indicating the presence of large particles over the diffraction limit. Incubation of the ternary complexes with serum slightly increased the size of the ternary complexes. Heat inactivation of serum tended to increase the number of large particles, consistent with the increase in PDI. Incubation of the ternary complexes with BSA in PBS increased the particle size compared with the control group. Incubation of the ternary complexes with PBS further increased the size and number of large particles. Large particles, approximately 10 μm in size, were observed in the PBS group. In addition, the particles after incubation with serum components were still spherical.

**Figure 8:**
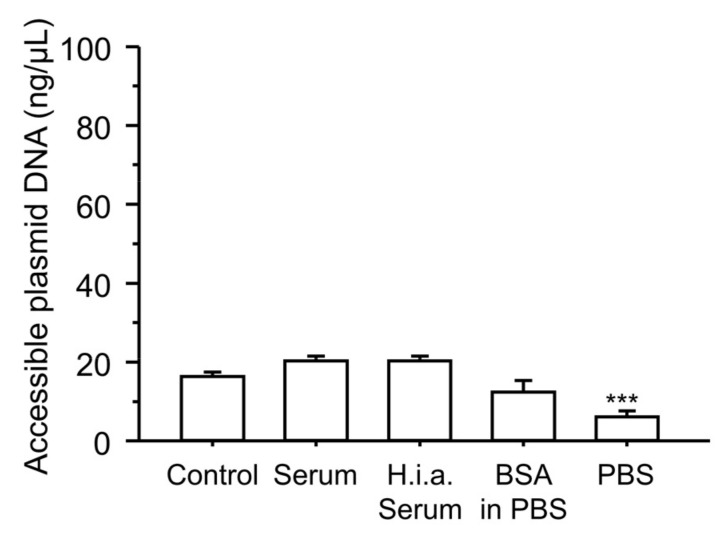
Effect of incubation with serum components on plasmid DNA accessibility in the γ-PGA/PEI/plasmid DNA ternary complexes. The ternary complexes were incubated with 5% glucose solution (control), serum, heat-inactivated serum (h.i.a serum), BSA in PBS, or PBS for 5 min at 37 °C, and then accessible plasmid DNA was quantified. The total amount of plasmid DNA in the complexes was 100 ng/μL. Each bar represents the mean + S.D. of 3 replicates. *** indicates statistical differences vs. control (*p* < 0.001).

To further evaluate the effect of serum components on the physicochemical properties of the ternary complexes, we analyzed the accessibility of plasmid DNA in the ternary complexes after incubating the complexes with serum components (Figure 8). In the control group (incubation with 5% glucose), 16% of the plasmid DNA in the complexes was accessible. Incubation with serum, heat-inactivated serum, or BSA in PBS did not significantly change plasmid DNA accessibility. In contrast, incubation of the ternary complexes with PBS significantly decreased the amount of accessible plasmid DNA.

## 4. Discussion

Tremendous efforts have been made to understand the mechanisms underlying gene transfer. These mechanisms include interactions with blood components, cellular association and uptake, endocytic routes, endosomal escape, cytosolic and nuclear transport, and dissociation of cargo from carriers [41]. In this study, we focused on the interaction of complexes with blood components as the first step in in vivo gene transfer.

The effects of interaction with serum on the gene expression were similar for both the binary and ternary complexes (Figure 1 and Figure 2). However, heat inactivation of serum produced different results for the binary and ternary complexes. The results for the binary complexes were similar to those for the cationic liposome/plasmid DNA complexes [39,42]. Heat-labile components such as complements would contribute to in vivo gene transfer. In the case of the cationic liposomes, the interaction between the cationic liposomes and fibronectin contributed to the enhanced gene expression in first-pass organs [39,42]. In this study, we administered the binary complexes intravenously; therefore, the first-pass organ was the lung. However, the gene expression was enhanced not only in the lung but also in the liver and spleen. Another possibility is the effect of interactions with the complements. In the case of the cationic liposomes, complement component C3 enhances the hepatic gene expression after intraportal administration [42]. It is still unclear whether electrostatic interactions occur between the binary complexes and fibronectin/complement component C3. In contrast, heat inactivation of serum did not diminish the enhancing effect of serum on the gene expression (Figure 2). Because the ζ-potential of the ternary complexes was strongly negative, the cationic components in serum might electrostatically interact with the ternary complexes. To elucidate the effect of electrostatic interactions with serum components, we used ion-exchange columns to deplete cationic and anionic components from serum. The depletion of cationic components from the serum using a cation-exchange column produced results different from those of the anionic component-depleted serum (Figure 3). Cationic components may have positive effects on the hepatic gene expression, whereas cationic components may have negative effects on the splenic gene expression. However, the depleted serum of cationic and anionic components still possessed a high transfection efficiency. Therefore, we analyzed the components that contributed to gene transfer and found that serum albumin and salts in the buffer contributed to the enhanced effects on the liver and spleen (Figure 4 and Figure 5). In the case of the lungs, incubation with serum albumin and PBS did not increase the gene expression; therefore, components other than albumin and salts likely contributed to the enhanced effects in the lungs.

Both serum albumin and salts are abundant in blood. The reason why incubation of the ternary complexes with albumin and salts before administration enhanced the gene expression remains unclear. The major difference between the control and incubation groups (BSA in PBS and PBS groups) was the presence or absence of erythrocytes during the interaction with albumin and salts. In the case of cationic binary complexes, such as PEI/plasmid DNA and cationic liposome/plasmid DNA complexes, incubation of the binary complexes with serum components before administration may inhibit electrostatic interactions with erythrocytes in vivo. However, the ζ-potentials of the ternary complexes were negative, and it was unlikely that the ternary complexes electrostatically interacted with negatively charged erythrocytes. One possible reason for the enhanced gene expression is the increase in particle size (Figure 6a and Figure 7), which may increase the tissue retention time owing to reduced Brownian motion. In addition to reduced Brownian motion, filtration by the reticuloendothelial system may increase accumulation in the liver and spleen. Microscopic images (Figure 7) indicated that the number of large particles increased owing to the interaction with serum components. This was consistent with the increase in the PDI (Figure 6a). Large particles might be advantageous for endosomal escape by ensuring the time before the fusion of endosomes with lysosomes. The increased gene expression in the spleen and liver might be related to the increased number of large particles. Such size effects have also been reported for cationic liposomes in vitro and in vivo [43,44,45,46]. Another possibility might be the decreased absolute values of ζ-potential by the incubation with serum components (Figure 6b); this weakens the electrostatic repulsion between the ternary complexes and cells. The addition of salts to complexes such as cationic liposome/plasmid DNA complexes changes their structures [47]. These structural changes may concertedly contribute to these enhancing effects. Plasmid DNA accessibility in the ternary complexes was decreased by incubation with PBS, whereas incubation with serum, heat-inactivated serum, or BSA in PBS did not significantly change the accessibility (Figure 8). Recently, we found that plasmid DNA accessibility was positively correlated with the transfection efficiency of different cationic liposome/plasmid DNA complexes prepared under various conditions, in terms of liposomal size and temperature [48]. Therefore, there was a discrepancy between the ternary complexes and cationic liposome/plasmid DNA complexes in terms of the relationship between plasmid DNA accessibility and transfection efficiency. Since incubation of the ternary complexes with serum components did not increase plasmid DNA accessibility, the stability of the ternary complexes in serum may be good. The good stability might partially contribute to the enhanced gene expression in the spleen and liver.

In this study, we analyzed the effects of serum components on in vivo gene transfer in normal mice. Gene therapy will be performed under diseased conditions; therefore, the roles of serum components under diseased conditions may differ from those under normal conditions. In the case of cationic liposomes, fibronectin contributes to the pulmonary gene expression in normal mice [39]. In contrast, incubation with serum albumin before administration greatly enhances the gene expression in the lungs, liver, and spleen of hepatitis mice [49]. This effect of serum albumin may be attributed to changes in body condition, such as a slightly lower pH than the physiological value. Therefore, it is preferable to investigate the effects of serum components on in vivo gene transfer under both diseased and normal conditions.

The availability of the technique of incubating the complexes with serum components to enhance in vivo gene transfer might be limited as follows. The use of autologous serum for incubation with the ternary complexes before administration is cumbersome. Serum albumin and PBS might be more suitable because of their ease of preparation. However, the sizes of the particles were larger than that of the serum group. The shapes of the ternary complexes after incubation with serum components were spherical (Figure 7). This feature might be preferable, especially for large particles, to prevent capillary embolization. However, the maximum size of the particles was observed near the erythrocytes. A reduction in particle size while maintaining the enhanced gene expression based on the adjustment of salt concentration may be possible. This possibility will be pursued in the near future.

Biomolecules, such as γ-PGA, have diverse applications [50]. In addition to DNA, siRNA and mRNA can also be delivered using γ-PGA [51,52,53]. Other anionic biomolecules include hyaluronic acid [54,55,56,57,58], polynucleotides [59], and chondroitin sulfate [60,61]. The effects of biological components, such as serum, may differ among biomolecules in addition to administration routes. To better understand the use of these biomolecules, it is necessary to elucidate the effects of biological components on their efficacy and biodistribution.

In conclusion, thermosensitive components in the serum may be responsible for the increased gene expression of PEI/plasmid DNA binary complexes in the liver and spleen. In contrast, salts in serum might alter γ-PGA/PEI/plasmid DNA ternary complex-mediated transfection, especially in the spleen. Changes in the physicochemical characteristics of the complexes may contribute to their gene-expression-enhancing effects. This study provides basic information for the further development of effective gene delivery carriers.

## Data Availability

Data obtained in this study will be available upon reasonable request from the corresponding authors.

## References

[1] Martínez-Puente D.H., Pérez-Trujillo J.J., Zavala-Flores L.M., García-García A., Villanueva-Olivo A., Rodríguez-Rocha H., Valdés J., Saucedo-Cárdenas O., Montes de Oca-Luna R., Loera-Arias M.J. (2022). Plasmid DNA for Therapeutic Applications in Cancer. Pharmaceutics.

[2] Grisch-Chan H.M., Schwank G., Harding C.O., Thöny B. (2019). State-of-the-Art 2019 on Gene Therapy for Phenylketonuria. Hum. Gene Ther..

[3] Ginn S.L., Amaya A.K., Alexander I.E., Edelstein M., Abedi M.R. (2018). Gene therapy clinical trials worldwide to 2017: An update. J Gene Med..

[4] Schnell M.A., Zhang Y., Tazelaar J., Gao G.P., Yu Q.C., Qian R., Chen S.J., Varnavski A.N., LeClair C., Raper S.E. (2001). Activation of innate immunity in nonhuman primates following intraportal administration of adenoviral vectors. Mol. Ther..

[5] Khatri A., Shelke R., Guan S., Somanathan S. (2022). Higher Seroprevalence of Anti-Adeno-Associated Viral Vector Neutralizing Antibodies Among Racial Minorities in the United States. Hum. Gene Ther..

[6] Gao X., Kim K.S., Liu D. (2007). Nonviral gene delivery: What we know and what is next. AAPS J..

[7] Boussif O., Lezoualc’h F., Zanta M.A., Mergny M.D., Scherman D., Demeneix B., Behr J.P. (1995). A versatile vector for gene and oligonucleotide transfer into cells in culture and in vivo: Polyethylenimine. Proc. Natl. Acad. Sci. USA.

[8] Zanta M.A., Boussif O., Adib A., Behr J.P. (1997). In vitro gene delivery to hepatocytes with galactosylated polyethylenimine. Bioconjug Chem..

[9] Godbey W.T., Wu K.K., Hirasaki G.J., Mikos A.G. (1999). Improved packing of poly(ethylenimine)/DNA complexes increases transfection effi-ciency. Gene Ther..

[10] Kunath K., von Harpe A., Fischer D., Petersen H., Bickel U., Voigt K., Kissel T. (2003). Low-molecular-weight polyethylenimine as a non-viral vector for DNA delivery: Comparison of physicochemical properties, transfection efficiency and in vivo distribution withhigh-molecular-weight polyethylenimine. J. Control Release.

[11] Oh Y.K., Suh D., Kim J.M., Choi H.G., Shin K., Ko J.J. (2002). Polyethylenimine-mediated cellular uptake, nucleus trafficking and expression of cytokine plasmid DNA. Gene Ther..

[12] Thomas M., Lu J.J., Ge Q., Zhang C., Chen J., Klibanov A.M. (2005). Full deacylation of polyethylenimine dramatically boosts its gene delivery ef-ficiency and specificity to mouse lung. Proc. Natl. Acad. Sci. USA.

[13] Gomez J.P., Pichon C., Midoux P. (2013). Ability of plasmid DNA complexed with histidinylated lPEI and lPEI to cross in vitro lung and muscle vascular endothelial barriers. Gene.

[14] Dabbaghi M., Hashemi K., Oskuee R.K., Afkhami-Goli A. (2022). Reverse relation between cytotoxicity and Polyethylenimine/DNA ratio, the effect of using HEPES-buffered saline (HBS) medium in gene delivery. Toxicol. In Vitro.

[15] Patel A.K., Kaczmarek J.C., Bose S., Kauffman K.J., Mir F., Heartlein M.W., DeRosa F., Langer R., Anderson D.G. (2019). Inhaled Nanoformulated mRNA Polyplexes for Protein Production in Lung Epithelium. Adv. Mater..

[16] Zeng M., Zhou D., Alshehri F., Lara-Sáez I., Lyu Y., Creagh-Flynn J., Xu Q., Sigen A., Zhang J., Wang W. (2019). Manipulation of Transgene Expression in Fibroblast Cells by a Multifunctional Linear-Branched Hybrid Poly(β-Amino Ester) Synthesized through an Oligomer Combination Approach. Nano Lett..

[17] Zhou D., Cutlar L., Gao Y., Wang W., O’Keeffe-Ahern J., McMahon S., Duarte B., Larcher F., Rodriguez B.J., Greiser U. (2016). The transition from linear to highly branched poly(β-amino ester)s: Branching matters for gene delivery. Sci. Adv..

[18] Perni S., Prokopovich P. (2017). Poly-beta-amino-esters nano-vehicles based drug delivery system for cartilage. Nanomedicine.

[19] Petkova A.I., Kubajewska I., Vaideanu A., Schätzlein A.G., Uchegbu I.F. (2022). Gene Targeting to the Cerebral Cortex Following Intranasal Administration of Polyplexes. Pharmaceutics.

[20] Rodolfo C., Eusébio D., Ventura C., Nunes R., Florindo H.F., Costa D., Sousa Â. (2021). Design of Experiments to Achieve an Efficient Chitosan-Based DNA Vaccine Delivery System. Pharmaceutics.

[21] Yu Z., Lu Y., Cao W., Aleem M.T., Liu J., Luo J., Yan R., Xu L., Song X., Li X. (2021). Nano DNA Vaccine Encoding Toxoplasma gondii Histone Deacetylase SIR2 Enhanced Protective Immunity in Mice. Pharmaceutics.

[22] Maiorano G., Guido C., Russo A., Giglio A., Rizzello L., Testini M., Cortese B., D’Amone S., Gigli G., Palamà I.E. (2022). Hybrid Polyelectrolyte Nanocomplexes for Non-Viral Gene Delivery with Favorable Efficacy and Safety Profile. Pharmaceutics.

[23] Zenze M., Daniels A., Singh M. (2023). Dendrimers as Modifiers of Inorganic Nanoparticles for Therapeutic Delivery in Cancer. Pharmaceutics.

[24] Srinageshwar B., Florendo M., Clark B., Johnson K., Munro N., Peruzzaro S., Antcliff A., Andrews M., Figacz A., Swanson D. (2020). A Mixed-Surface Polyamidoamine Dendrimer for In Vitro and In Vivo Delivery of Large Plasmids. Pharmaceutics.

[25] Eliyahu H., Servel N., Domb A.J., Barenholz Y. (2002). Lipoplex-induced hemagglutination: Potential involvement in intravenous gene delivery. Gene Ther..

[26] Dekie L., Toncheva V., Dubruel P., Schacht E.H., Barrett L., Seymour L.W. (2000). Poly-L-glutamic acid derivatives as vectors for gene therapy. J. Control Release.

[27] Godbey W.T., Wu K.K., Mikos A.G. (1999). Poly(ethylenimine) and its role in gene delivery. J. Control Release.

[28] Goula D., Remy J.S., Erbacher P., Wasowicz M., Levi G., Abdallah B., Demeneix B.A. (1998). Size, diffusibility and transfection performance of linear PEI/DNA complexes in the mouse central nervous system. Gene Ther..

[29] Fischer D., Bieber T., Li Y., Elsässer H.P., Kissel T. (1999). A novel non-viral vector for DNA delivery based on low molecular weight, branched polyethylenimine: Effect of molecular weight on transfection efficiency and cytotoxicity. Pharm. Res..

[30] Oh Y.K., Kim J.P., Yoon H., Kim J.M., Yang J.S., Kim C.K. (2001). Prolonged organ retention and safety of plasmid DNA administered in polyethylenimine complexes. Gene Ther..

[31] Trubetskoy V.S., Wong S.C., Subbotin V., Budker V.G., Loomis A., Hagstrom J.E., Wolff J.A. (2003). Recharging cationic DNA complexes with highly charged polyanions for in vitro and in vivo gene delivery. Gene Ther..

[32] Ito T., Iida-Tanaka N., Koyama Y. (2008). Efficient in vivo gene transfection by stable DNA/PEI complexes coated by hyaluronic acid. J. Drug Target..

[33] Orson F.M., Song L., Gautam A., Densmore C.L., Bhogal B.S., Kinsey B.M. (2002). Gene delivery to the lung using pro-tein/polyethylenimine/plasmid complexes. Gene Ther..

[34] Kurosaki T., Kitahara T., Fumoto S., Nishida K., Nakamura J., Niidome T., Kodama Y., Nakagawa H., To H., Sasaki H. (2009). Ternary complexes of pDNA, polyethylenimine, and gamma-polyglutamic acid for gene delivery systems. Biomaterials.

[35] Kurosaki T., Kitahara T., Kawakami S., Higuchi Y., Yamaguchi A., Nakagawa H., Kodama Y., Hamamoto T., Hashida M., Sasaki H. (2010). Gamma-polyglutamic acid-coated vectors for effective and safe gene therapy. J. Control Release.

[36] Sakurai F., Nishioka T., Saito H., Baba T., Okuda A., Matsumoto O., Taga T., Yamashita F., Takakura Y., Hashida M. (2001). Interaction between DNA-cationic liposome complexes and erythrocytes is an important factor in systemic gene transfer via the intravenous route in mice: The role of the neutral helper lipid. Gene Ther..

[37] Yang J.P., Huang L. (1997). Overcoming the inhibitory effect of serum on lipofection by increasing the charge ratio of cationic liposome to DNA. Gene Ther..

[38] Zhu D., Yan H., Zhou Z., Tang J., Liu X., Hartmann R., Parak W.J., Feliu N., Shen Y. (2018). Detailed investigation on how the protein corona modulates the physicochemical properties and gene delivery of polyethylenimine (PEI) polyplexes. Biomater. Sci..

[39] Yoshikawa N., Fumoto S., Nakashima M., Shimokawa K., Miyamoto H., Nishida K. (2013). The role of fibronectin in pulmonary gene transfer following intravenous administration of lipoplex in mice. Biol. Pharm. Bull..

[40] Lausted C., Hu Z., Hood L. (2008). Quantitative serum proteomics from surface plasmon resonance imaging. Mol. Cell. Proteom..

[41] Fumoto S., Yamamoto T., Okami K., Maemura Y., Terada C., Yamayoshi A., Nishida K. (2021). Understanding In Vivo Fate of Nucleic Acid and Gene Medicines for the Rational Design of Drugs. Pharmaceutics.

[42] Yoshikawa N., Sakamoto K., Mizuno S., Sakaguchi J., Miyamoto H., Mine T., Sasaki H., Fumoto S., Nishida K. (2011). Multiple components in serum contribute to hepatic transgene expression by lipoplex in mice. J. Gene Med..

[43] Almofti M.R., Harashima H., Shinohara Y., Almofti A., Li W., Kiwada H. (2003). Lipoplex size determines lipofection efficiency with or without serum. Mol. Membr. Biol..

[44] Caracciolo G., Callipo L., De Sanctis S.C., Cavaliere C., Pozzi D., Laganà A. (2010). Surface adsorption of protein corona controls the cell internali-zation mechanism of DC-Chol-DOPE/DNA lipoplexes in serum. Biochim. Biophys. Acta.

[45] Prabha S., Arya G., Chandra R., Ahmed B., Nimesh S. (2016). Effect of size on biological properties of nanoparticles employed in gene delivery. Artif. Cells Nanomed Biotechnol..

[46] Ogris M., Steinlein P., Kursa M., Mechtler K., Kircheis R., Wagner E. (1998). The size of DNA/transferrin-PEI complexes is an important factor for gene expression in cultured cells. Gene Ther..

[47] Fumoto S., Kawakami S., Ito Y., Shigeta K., Yamashita F., Hashida M. (2004). Enhanced hepatocyte-selective in vivo gene expression by stabilized galactosylated liposome/plasmid DNA complex using sodium chloride for complex formation. Mol. Ther..

[48] Hu D., Fumoto S., Yoshikawa N., Peng J., Miyamoto H., Tanaka M., Nishida K. (2023). Diffusion coefficient of cationic liposomes during lipoplex formation determines transfection efficiency in HepG2 cells. Int. J. Pharm..

[49] Yoshikawa N., Fumoto S., Yoshikawa K., Hu D., Okami K., Kato R., Nakashima M., Miyamoto H., Nishida K. (2020). Interaction of Lipoplex with Albumin Enhances Gene Expression in Hepatitis Mice. Pharmaceutics.

[50] Li J., Li C., Zhao Z., Guo Y., Chen H., Liu P., Zhao M., Guo J. (2024). Biomolecules meet organic frameworks: From synthesis strategies to diverse applications. Nanoscale.

[51] Peng S.F., Hsu H.K., Lin C.C., Cheng Y.M., Hsu K.H. (2017). Novel PEI/Poly-γ-Gutamic Acid Nanoparticles for High Efficient siRNA and Plasmid DNA Co-Delivery. Molecules.

[52] Zhang H., Gao X., Sun Q., Dong X., Zhu Z., Yang C. (2024). Incorporation of poly(γ-glutamic acid) in lipid nanoparticles for enhanced mRNA delivery efficiency in vitro and in vivo. Acta Biomater..

[53] Dasgerdi N.K., Gumus N., Bayraktutan H., Jackson D., Polra K., McKay P.F., Atyabi F., Dinarvand R., Shattock R.J., Martinez-Pomares L. (2024). Charge neutralized poly(β-amino ester) polyplex nanoparticles for delivery of self-amplifying RNA. Nanoscale Adv..

[54] Aldawsari H.M., Dhaliwal H.K., Aljaeid B.M., Alhakamy N.A., Banjar Z.M., Amiji M.M. (2019). Optimization of the Conditions for Plasmid DNA Delivery and Transfection with Self-Assembled Hyaluronic Acid-Based Nanoparticles. Mol. Pharm..

[55] Zhao M., Zhang M., Yu Q., Fei W., Li T., Zhu L., Yao Y., Zheng C., Zhang X. (2022). Hyaluronic Acid-Modified Nanoplatforms as a Vector for Targeted Delivery of Autophagy-Related Gene to the Endometriotic Lesions in Mice. Front. Bioeng. Biotechnol..

[56] Vicente-Pascual M., Gómez-Aguado I., Rodríguez-Castejón J., Rodríguez-Gascón A., Muntoni E., Battaglia L., Del Pozo-Rodríguez A., Solinís Aspiazu M.Á. (2020). Topical Administration of SLN-Based Gene Therapy for the Treatment of Corneal Inflammation by De Novo IL-10 Production. Pharmaceutics.

[57] Ebrahimian M., Hashemi M., Farzadnia M., Zarei-Ghanavati S., Malaekeh-Nikouei B. (2022). Development of targeted gene delivery system based on liposome and PAMAM dendrimer functionalized with hyaluronic acid and TAT peptide: In vitro and in vivo studies. Biotechnol. Prog..

[58] Nabar N., Dacoba T.G., Covarrubias G., Romero-Cruz D., Hammond P.T. (2024). Electrostatic adsorption of polyanions onto lipid nanoparticles controls uptake, trafficking, and transfection of RNA and DNA therapies. Proc. Natl. Acad. Sci. USA.

[59] Kodama Y., Ohkubo C., Kurosaki T., Egashira K., Sato K., Fumoto S., Nishida K., Higuchi N., Kitahara T., Nakamura T. (2015). Secure and effective gene delivery system of plasmid DNA coated by polynucleotide. J. Drug Target..

[60] Ito T., Sugiura K., Hasegawa A., Ouchi W., Yoshimoto T., Mizoguchi I., Inaba T., Hamada K., Eriguchi M., Koyama Y. (2021). Microbial An-tigen-Presenting Extracellular Vesicles Derived from Genetically Modified Tumor Cells Promote Antitumor Activity of Den-dritic Cells. Pharmaceutics.

[61] Lin W.J., Lee W.C. (2018). Polysaccharide-modified nanoparticles with intelligent CD44 receptor targeting ability for gene delivery. Int. J. Nanomed..

